# Nuclear Receptor Subfamily 2 Group F Member 1a (nr2f1a) Is Required for Vascular Development in Zebrafish

**DOI:** 10.1371/journal.pone.0105939

**Published:** 2014-08-26

**Authors:** Bao-Jueng Wu, Chien-Chih Chiu, Chun-Lin Chen, Wen-Der Wang, Jia-Hong Wang, Zhi-Hong Wen, Wangta Liu, Hsueh-Wei Chang, Chang-Yi Wu

**Affiliations:** 1 Department of Biological Sciences, National Sun Yat-sen University, Kaohsiung, Taiwan; 2 Marine Biotechnology Doctoral Program, National Sun Yat-sen University, Kaohsiung, Taiwan; 3 Department of Marine Biotechnology and Resources, National Sun Yat-sen University, Kaohsiung, Taiwan; 4 Zuoying Branch of Kaohsiung Armed Forces General Hospital, Kaohsiung, Taiwan; 5 Department of Biotechnology, Kaohsiung Medical University, Kaohsiung, Taiwan; 6 Department of Bioagricultural Science, National Chiayi University, Chiayi, Taiwan; 7 Department of Biomedical Science and Environmental Biology, Kaohsiung Medical University, Kaohsiung, Taiwan; Institute of Cellular and Organismic Biology, Taiwan

## Abstract

Genetic regulators and signaling pathways are important for the formation of blood vessels. Transcription factors controlling vein identity, intersegmental vessels (ISV) growth and caudal vein plexus (CVP) formation in zebrafish are little understood as yet. Here, we show the importance of the nuclear receptor subfamily member 1A (nr2f1a) in zebrafish vascular development. Amino acid sequence alignment and phylogenetic analysis of nr2f1a is highly conserved among the vertebrates. Our in situ hybridization results showed *nr2f1a* mRNA is expressed in the lateral plate mesoderm at 18 somite stage and in vessels at 24–30 hpf, suggesting its roles in vasculization. Consistent with this morpholino-based knockdown of *nr2fla* impaired ISV growth and failed to develop fenestrated vascular structure in CVP, suggesting that *nr2f1a* has important roles in controlling ISV and CVP growth. Consequently, *nr2f1a* morphants showed pericardial edema and circulation defects. We further demonstrated reduced ISV cells and decreased CVP endothelial cells sprouting in *nr2f1a* morphants, indicating the growth impairment of ISV and CVP is due to a decrease of cell proliferation and migration, but not results from cell death in endothelial cells after morpholino knockdown. To test molecular mechanisms and signals that are associated with *nr2f1a*, we examined the expression of vascular markers. We found that a loss of *nr2f1a* results in a decreased expression of vein/ISV specific markers, *flt4, mrc1*, vascular markers *stabilin* and *ephrinb2.* This indicates the regulatory role of nr2f1a in controlling vascular development. We further showed that *nr2f1a* likely interact with *Notch* signaling by examining *nr2f1a* expression in *rbpsuh* morphants and DAPT-treatment embryos. Together, we show nr2f1a plays a critical role for vascular development in zebrafish.

## Introduction

Vertebrate blood vessels are critical for nutrient and oxygen supply and are developed in an evolutionarily conserved manner. The arteries and veins are formed from angioblast progenitors during vasculogenesis. After the establishment of these vessels, additional vessels develop from pre-existing vessels by angiogenesis. This process includes sprouting, migration, lumenization, branching and fusion to form a coordinated pattern of the vessel plexus, which is tightly controlled by growth factors, regulators and multiple signaling pathways. Ultimately, transcription factors play a central role in the regulation of the vascular development after receiving signals [1]–[5].

Zebrafish have been shown as a suitable vertebrate model to study developmental processes – so in vascular development [6]. In zebrafish, the dorsal aorta (DA) and posterior cardinal vein (PCV) form a primitive circulatory loop during vasculogenesis, then sprouting angiogenesis from the DA to form intersegmental vessels (ISVs) and from the axial vein to develop a honeycomb-like network called caudal vein plexus (CVP) via distinct signalling pathways. Specifically, the development of the ISV of the trunk begins with an angioblast sprouting from the DA and dorsad migration along the somite boundaries towards the midline where it undergoes a cell division. One daughter cell continues to migrate dorsally until it reaches the dorsal aspect of the embryo and fuses with angioblasts from anterior and posterior ISVs to form the dorsal longitudinal anastomotic vessels (DLAVs). The leading cells to migrate from the vessel are called tip cells. These are proliferative and show multiple filopodia, while the less proliferative, stationary cells which lumenize behind the tip cell are called stalk cells [7]. This process is largely controlled by VEGF and Notch signalling. However, Wiley and co-workers showed recently that BMP signalling pathways regulate sprouting angiogenesis from the axial vein to form CVP, which provides a distinct mechanism of angiogenic sprouting [8]. This processing is mediated by Dab2 to promote internalization of the BMP2 signaling complex and modulates SMAD phosphorylation [9]. In spite of this, knowledge related to molecules that are important for CVP angiogenesis is still limited. A complex coordination among these signaling pathways have a critical role in controlling endothelial tip cell fates during angiogenesis. Concerning the responsible factors, the orphan nuclear receptor chicken ovalbumin upstream promoter transcription factor II (CoupTFII; a.k.a. nr2f2) has been shown function in angiogenesis mediated upregulation of Angiopoietin-1 in mice [10], [11]. However, there is still limited understanding about the transcription factors that control the tip cell fate. In addition, the angiogenic function of nr2f2 is not very clear in zebrafish based on our study and recent discoveries [12], [13].

Studies of mNR2F (CoupTF) family have been demonstrated their physiological and pathologic importance. mNr2f2 have been identified their function in cell-fate specification, organogenesis, angiogenesis, lymphagenesis, metabolism and a variety of diseases [14]. In the field of mouse vascular development, mNr2f2 is a major determinant of venous identity and involved in angiogenesis [11]. In addition, mNr2f2 physically and functionally interact with Prox1 to specific lymphatic endothelial fate and promote the formation of lymphatic vasculature [15]. Our unpublished data also showed that nr2f2 in zebrafish plays a minor role in venous identity and functions critically in lymphangiogenesis, which is similar to recent reports [12], [13]. On the other hand, mammalian Nr2f1 has been showed as a critical regulator of CNS and peripheral nervous system development and controls cell differentiation in the inner ear [16], [17]. However, there is no document shown that mNr2f1 functions in vasculature.

In this study, we hypothesize the conserved NR2F family has an important function in zebrafish blood vessel formation. We showed *nr2f1a* mRNA is expressed in developing vessels, suggesting its roles in vasculature. We also showed loss of *nr2f1a* impairs ISV growth and CVP patterning. The impairment of vascular development results in pericardial edema and circulation defects at later developmental stage. We further demonstrated loss of *nr2f1a* results in a decreased expression of vascular specific markers, *flt4, mrc1, ephrinb2* and *stabilin*, suggested the regulatory role of *nr2f1a* in controlling vascular development. Finally, we showed that *nr2f1a* likely interact with the *Notch* signalling. Together, we show nr2f1a plays a critical role for vascular development in zebrafish.

## Materials and Methods

### Zebrafish strains and husbandry

Zebrafish (*Danio rerio*) wild-type (AB) embryos and transgenic lines: *Tg(kdrl:eGFP)^la116^*, *Tg(gata:dsRed)* and *Tg(fli1a:negfp)^y7^* [18] from Taiwan Zebrafish Core Facility at Academia Sinica or NTHU-NHRI were raised and maintained at the 28.5°C incubator according to The Zebrafish Book [19] with approval from the National Sun Yat-sen University Animal Care Committee. Embryos were treated with 0.003% 1-Phenyl-2-Thiourea (PTU) (Sigma, St. Louis, MO) at six hours post fertilization (hpf) to prevent pigment formation.

### Morpholino and Tol2 DNA construct injections

Morpholinos for each gene were obtained from Gene-Tools, LLC (Philomath, OR), resuspended in ddH_2_O to 2 mM stock and further diluted to the working concentration with 0.5% phenol red (Sigma). To knockdown *nr2f1a* gene expression, we used *nr2f1a*-ATGMO (5′-CCAGACGCTAACTACCATTGCCATA-3′) to block translation and *nr2f1a*-e2i2MO (5′-GCCTCGTCTCACACACTCACCTGAC-3′) targeting the boundary of exon 2 and inton 2 to interfere with its splicing. Other morpholinos used in the paper are Rbpsuh ORFMO 5′- CAAACTTCCCTGTCACAACAGGCGC-3′ [2]. Microinjections were carried out according to Larmont et al [20]. Briefly, embryos were immobilized in an injection tray with 3% agar plate. MOs or expression vectors were injected into 1-2-cell-stage embryos. After injection, embryos were cultured in E3 buffer until being examined. To generate *fli1:nr2f1a* overexpressing embryos, we used the Tol2kit vectors with multisite Gateway cloning system (Invitrogen) to build expression constructs [21]. nr2f1a coding region franking with *attb1/b2* sequences was amplified from cDNA using primers nr2f1a-f1-attb1: 5′-GGGGACAAGTTTGTACAAAAAAGCAGGCTCCACCATGGCAATGGTAGTTAGCGTC-3′ and nr2f1a-r1-attb2:

5′-GGGGACCACTTTGTACAAGAAAGCTGGGTTTCATTGAATGGACATGTAGGG-3′. A Gateway compatible 0.8 kb *fli1* promoter has been described [30] Approximately 100 pg of plasmid DNA was co-injected with 40 pg of Tol2 mRNA into 1-cell embryos. Success of transgenesis can be verified in *Tg(fli1:nr2f1a)* transient transgenic, overexpressing embryos by expression of *cmlc* (cardiomyocyte light chain) driven GFP from the vector backbone.

### Morpholino efficiency

The efficiency of morpholinos that cause mis-splicing was determined using reverse-transcriptase polymerase chain reaction (RT-PCR) with primers spanning either side of the targeted exon. RNAs from both injected and uninjected control were collected embryos using RNeasy mini kit (Qiagen). Single strand cDNA was synthesized with Re-Transcription kit (Roche) with reverse transcriptase and oligo-dT primer according to the manufacturer’s instructions. For *nr2f1a* MO efficiency and specificity test, primers nr2f1a_MO_f 5′-CTGCCTATTGACCAACACC-3′ and nr2f1a_MO_r 5′-CTCTCGATGTGTGCAGCATC-3′ were used, while for GAPDH (a loading control), primers GAPDH_F 5′- TGCTGTAACCGAACTCATTGTC-3′ and GAPDH_R 5′- CAAGCTTACTGGTATGGCCTTC-3′ were used. The results were visualized by running 5 µl of the PCR reaction on 1.0% agarose gel.

### Whole-mount in situ hybridization

Whole-mount in situ hybridization was performed as described in [22], [23]. Probes for *nr2f1a* was obtained by PCR using primers described in Table S1 in File S1 and in vitro transcription using T7 Polymerase (Roche) with DIG-labeled UTP. The *flt4, mrc1, stabilin,* and *ephrinb2a* probes have been described [2], [24]. Embryos will be fixed using 4% paraformaldehyde and stored at −20°C in methanol for up to 6 months. After rehydration, permeabilization in 10 µg/mL Proteinase K, the probe against *target* mRNA will be added to embryos in hybridization buffer (Hyb) at 65°C overnight, then washed by 75%Hyb, 50%Hyb, 25%Hyb in 2X sodium citrate buffer (SSC) and 0.2X SSC for 1 hour. After blocking with 1%BSA in maleic acid buffer, an AP-conjugated anti-Dig antibody will be added and proceeded to react with NBT/BCIP substrate (Roche). The reaction was stopped by fixing for 15 minutes in 4% PFA, followed by two washes in PBT. Embryos were embedded in 3% methylcellulose and photographed.

### Image and quantification

Whole mount embryos were mounted in 3% methyl cellulose (Sigma) or 1.5% low melt agarose (Invitrogen). White light or fluorescent images were photographed on a Zeiss Lumar V12 stereomicroscope equipped with an AxioCam HRc camera and AxioVision software (Carl Zeiss). Final figures were made using Adobe Photoshop. Confocal images were collected on a Zeiss LSM700 confocal microscope, and presented as maximal intensity projections generated in ImageJ (NIH, Bethesda, Maryland, USA). Embryos were immobilized and embedded in 5% Tricaine in 0.5% low melting point agarose (Invitrogen) immediately before the first time point of interest. For imaging at multiple time points, embryos were left embedded in 0.5% LMP agarose but were covered with E3 solution. This removed the paralyzing effect of Tricaine while ensuring the general position of the embryo did not change between time points. For cryosection, embryos were fixed with tissue freezing medium, Tek OCT Compound and sectioned at 10 µM using a Leica CM3050S cryostat and photographed on IX71 inverted microscope (Olympus) with an SPOT RT3 camera (DIAGNOSTIC Inc., Sterling heights, MI). The number of cells in the ISVs was determined by counting from individual slices of confocal stacks. The counting area is between 5–15 ISVs in the yolk extension. CVP loop number is counting the honeycomb structure from the end of yolk extension to the end of tail.

### TUNEL staining

Embryos were fixed in 4% paraformaldehyde (PFA) and stored at −20°C in methanol. Embryos were washed with gradients of methanol/PBT starting with 3∶1 methanol:PBT (1X phosphate buffered saline, 0.1% Tween-20) and ending with 100% PBT. After digestion in 10 µg/mL Proteinase K for 20 minutes, embryos were fixed in 4% PFA for 15 minutes, then treated with 3% Hydrogen Peroxide in 0.1% PBT for 1 hour at room temperature in the dark and washed with PBT to eliminate endogenous peroxidase. For each sample group, 45 µl of TUNEL (TdT-mediated dUTP-X nick end labeling) label was pre-mixed with 5 µl of TUNEL enzyme and added to the embryos for 3 hours at 37°C in the dark. As a control, 50 µl of TUNEL label solution without enzyme was used. Embryos washed with PBT to remove unincorporated nucleotides and endogenous antibodies were blocked with 5% Normal Sheep Serum (NSS) in PBT for 2 hours at room temperature, then incubated with peroxidase (POD) conjugated anti-fluorescein antibody diluted 1∶2000 (Roche) in 5% NSS/PBT over night at 4°C in the dark, washed four times in PBT, and visualized using DAB kits (Roche).

### RNA extraction and quantitative real-time PCR (qRT-PCR)

Total RNA was purified using the RNeasy Mini kit (Qiagen). mRNA was reverse transcribed to cDNA using Reverse Transcriptase and oligo-dT primer (Roche) according to the manufacturer’s instructions. Quantitative RT-PCR was performed using a LightCycle 96 instrument (Roche) with SYBR Green I Master (Roche). qRT-PCR primers are listed in Table S1 in File S1. Relative gene expression levels were analyzed by the ΔΔ C_t_ method, with elongation factor 1α (EF1α) as a reference gene. All reactions were performed as biological triplicates.

### DAPT treatment

A stock solution of 25 mM DAPT (Sigma) dissolved in DMSO was diluted to 75 µM for the working concentration and added to embryos E3 medium at 6 hpf. Control embryos were treated with an equivalent dose 0.3% of DMSO.

## Results

### Zebrafish nr2f1a is conserved among vertebrates and close to mammalian Nr2f2

The NR2F family consists of two highly homologous subtypes, nr2F1 and nr2F2. Pereira et al. showed that mammalian Nr2f2 (CoupTFII) is required for angiogenesis and heart development mediated by downregulating a proangiogenic soluble factor angiopoietin-1 [11]. You et al. showed that loss of mNr2f2 in mouse leads to an almost complete loss of the cardinal vein [10]. However, morpholino knockdown of zebrafish nr2f2 in embryos leads to a reduction in venous marker expression without gross vein and ISV anomalies [12] and our unpublished results. Thus, we went back to examine the phylogenetic relationship of nr2f1/2 in zebrafish and other species.

**Figure 1:**
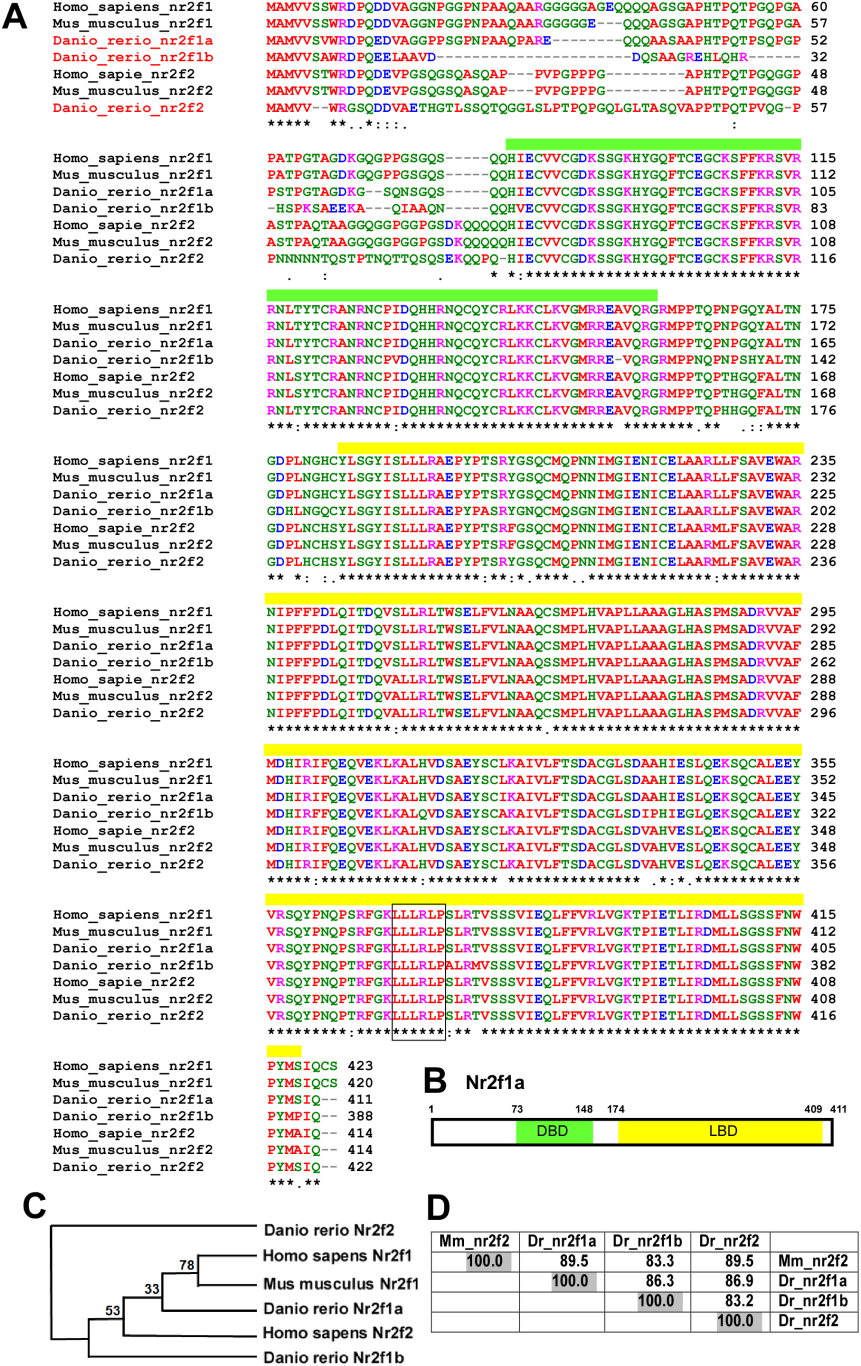
nr2f1a is conserved among vertebrates. (**A**) Comparison of the amino acid sequence of zebrafish nr2fIa to orthologs from other species by using ClustalW2 software. Identical amino acids are marked (*) below the sequence. The yellow bar represents a putative ligand binding domain and green box area indicates a zinc-finger DNA binding domain. Squared box indicates a conserved LLLRLP motif. (**B**) Schematic drawing shows that zebrafish nr2f1a protein contains 411 amino acids with the DNA binding domain (DBD) and ligand binding domain (LBD). (**C**) Cladograms provided from phylogenetic analysis of nr2f1a orthologs in different species using ClustalW2-phylogeny with the neighbor-joining method. The number (bootstrap values) at the nodes represent the possibility of groupings between 2 different nr2f members from different species. (**D**) Percent amino acid identity matrix among mouse (*Mus_musculus*) Nr2f2 and zebrafish (*Danio_rerio*) *nr2f1a, nr2f1b* and *nr2f2.*

We started to analyse the amino acid sequences among those vertebrates, human, mouse and zebrafish. By comparing amino acid sequences of zebrafish nr2f1a with orthologs from other species, highly identical amino acids are showed (>83%) (Figure 1A, D). We also identify the putative ligand binding domain (similarity = 95%), a zinc-finger DNA binding domain (similarity = 98%) and a protein-protein interaction LLLRLP motif (Figure 1B). Those suggest the function of zebrafish nr2f1a as nuclear transcriptional factor and may play similar functions as mammalian Nr2f1/2. Phylogenetic analysis of nr2f1a orthologs in different species suggests the high evolutionary conservation of NR2F family members in different species (Figure 1C). Since mouse Nr2f2 showed significant vascular functions, we focus on the comparison of conservation among mouse nr2f2 and zebrafish nr2f1a, nr2f1b and nr2f2. As shown in Figure 1D, mouse Nr2f2 are highly conserved with zebrafish nr2f2, nr2f1a (both have score of 89.5) and nr2f1b (score of 83.3). Since our previous nr2f2 study in zebrafish showed only minor effects in vasculature, we hypothesize that nr2f1a may play important functions in vascularization similar to the mammalian Nr2f2 (CoupTFII).

### *nr2f1a* is expressed in vasculature during development

**Figure 2:**
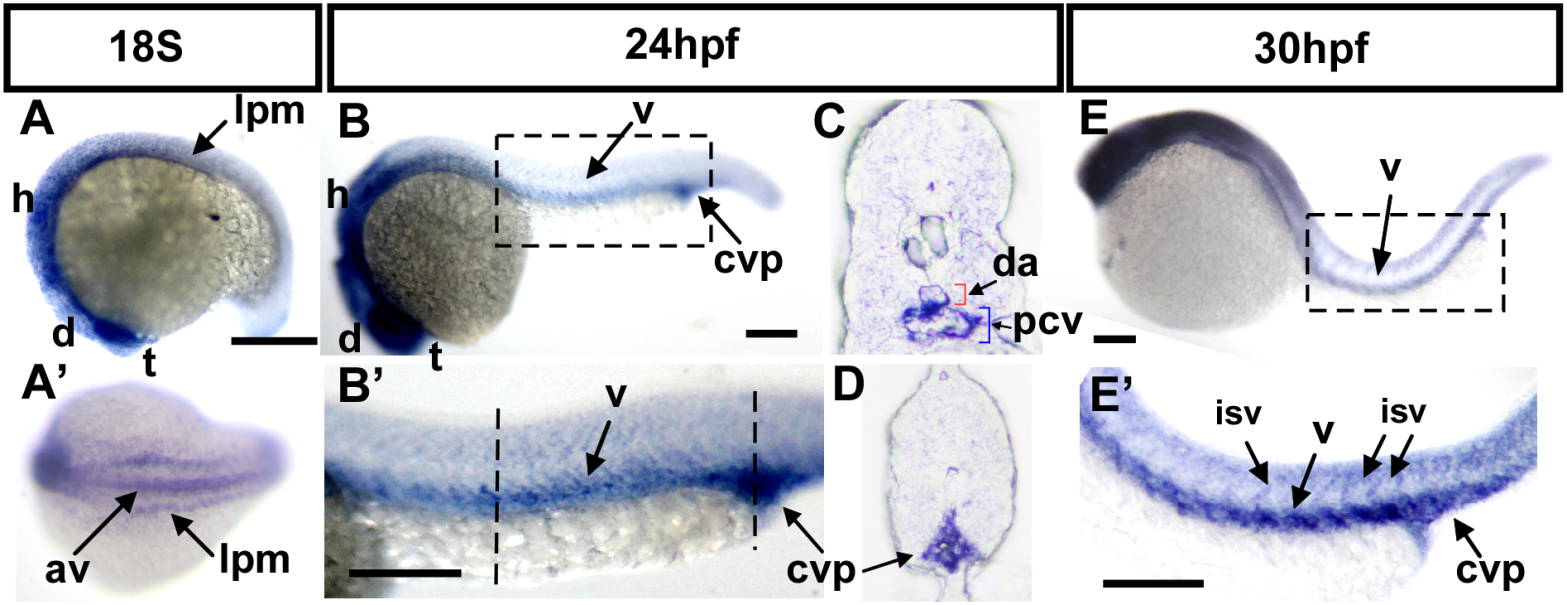
Spatiotemporal expression of *nr2f1a* during development. (**A**) *nr2f1a* expression can be observed at the 18S stage in the lateral plate mesoderm (lpm), the telencephalon (t), diencephalon (d) and hindbrain (h). (**A’**) Dorsal view of embryos show that *nr2f1a* is expressed at lpm and the axial vessels (av). (**B, B’**) At 24 hpf, *nr2f1a* is expressed in the telencephalon (t), diencephalon (d), hindbrain (h), as well as in vessels (v), and caudal vein plexus (CVP) of the trunk. **B’** is an enlargement of **B**. (**C, D**) Cross sections of embryos from B’ show that nr2f1a is expressed in dorsal aorta (da), posterior cardinal vein (pcv), and caudal vein plexus (CVP). (**E, E’**) At 30 hpf, *nr2f1a* expression continues in the head, vessels (v), intersegmental vessels (ISV) and caudal vein plexus (CVP) of the trunk. **E’** is an enlargement of **E**. Scale bars in all figures represent 100 µm.

To examine the potential role of nr2f1a in vascular development, we first determined its expression by whole-mount in sit hybridization during zebrafish embryonic development. At the 18 somite stage (S), *nr2f1a* is expressed in the lateral plate mesoderm (lpm), the telencephalon (t), diencephalon (d) and hindbrain (h). (Figure 2A). A dorsal view of embryos showed *nr2f1a* being expressed at lpm and axial vessels (Figure 2A’). Expression at the lateral plate mesoderm is corresponding to the location in the developing vasculature. At 24 hpf, *nr2f1a* is expressed in the telencephalon, diencephalon, hindbrain, as well as vessels of the trunk and caudal vein plexus (CVP) (Figure 2B, B’). Cross-sections of embryos show that *nr2f1a* is expressed in the dorsal aorta (da), posterior cardinal vein (pcv) and caudal vein plexus (CVP). At 30 hpf, *nr2f1a* expression continues in the head, vessels (v), intersegmental vessels (ISV) of the trunk and caudal vein plexus (CVP) (Figure 2E, E’). Those data show the expression of *nr2f1a* in vasculature during embryonic development and suggests the role of *nr2f1a* somewhere in vascular development. The expression pattern of *nr2f1a* in the head region from our study is similar to the previous studies by Bertrand et al and Love et al [25], [26] but the expression in vasculature is not described in previous publications. We found the expression level in the vasculature is much lower than in the head region. Thus, with shorter reaction time for in-situ staining, we can see clear expression pattern of *nr2f1a* in the head (data not shown) but no signals in the vasculature. Moreover, our *nr2f1a* expression is gene-specific because its expression shows different pattern from *nr2f1b* and *nr2f2* compared to the previous studies [25], [26] and ZFIN database [27].

### Knockdown of *nr2f1a* results in vascular defects

**Figure 3:**
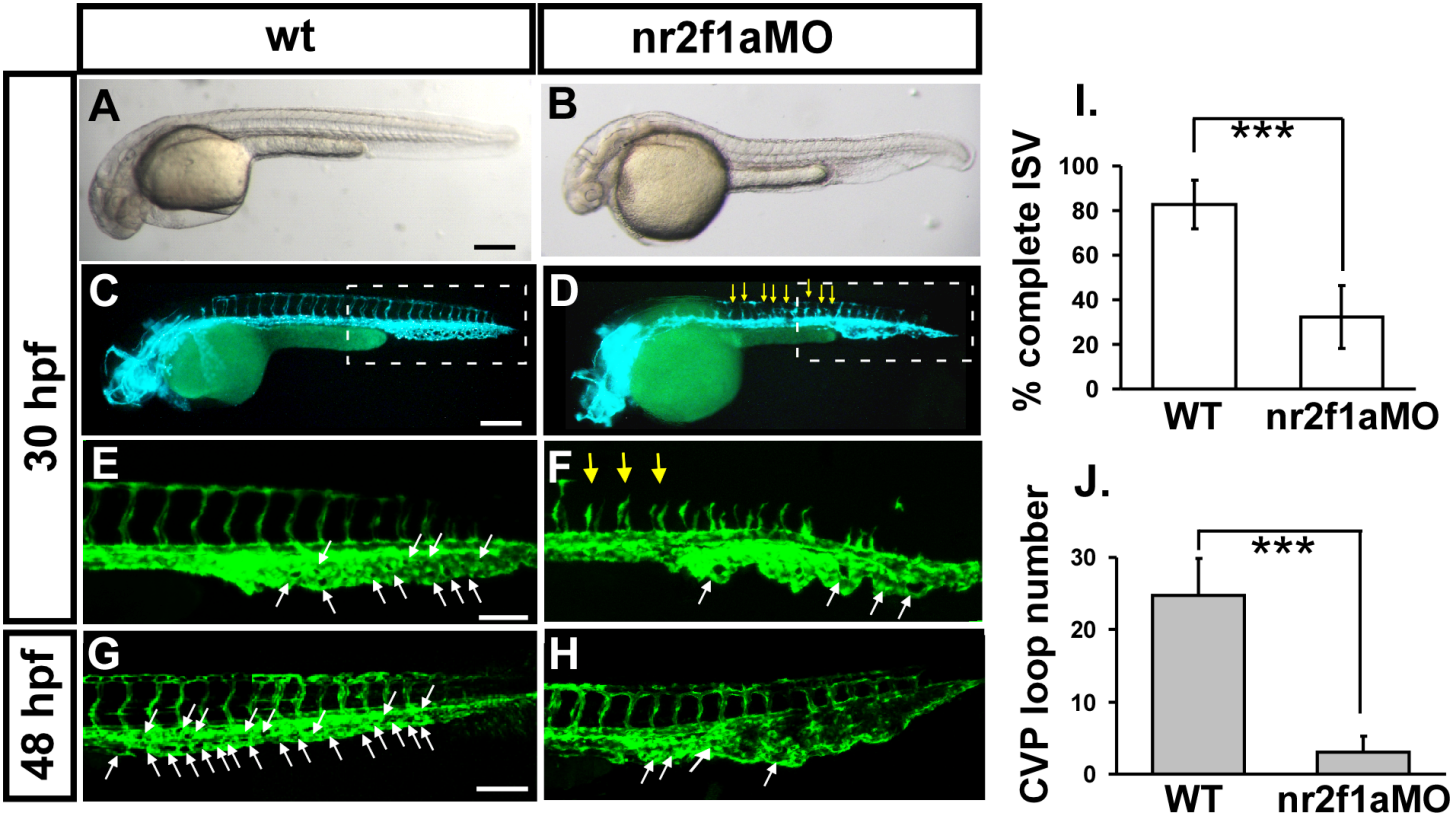
Knockdown of nr2f1a causes defects in zebrafish vascular development. (**A–H**) Loss of *nr2f1a* showing ISV growth defect (yellow arrows in **F**) and mis-pattern plexus at the CVP (caudal vein plexus) (white arrows) compared to wild-type control (**E**) at 30 hpf. **E** and **F** is enlargement of **C** and **D**, respectively. At 48 hpf, the swallowed plexus becomes obvious (**H**). At 30 hpf, in uninjected control embryos, intersegmental vessels (isv) have reached the DLAV at the dorsal aspect of the embryo (**C, E**) and the caudal vein plexus (cvp) formed honeycomb-like structures at the tail (**E,** white arrows). At the same stage ISVs are stalled at mid-somite in nr2f1a^e2i2^ morphants (**D, F**). (**I**) Quantification of percentage of completed ISV shows a ∼50% increase compared to *nr2f1a* morphants (n = 26 in wt and n = 34 in nr2f1aMO) at 30 hpf. (**J**) Quantification of loop formation at CVP shows a 4-fold decreased in *nr2f1a* morphants (n = 10 in wt and in nr2f1aMO) at 48 hpf. (*** refers to p<0.0001 by an unpaired student’s t-test. Scale bars are 200 µm for A–D, and 100 µm for E–H.

To identify the functional role of *nr2f1a* in vascular development, we knocked down its expression in *Tg (kdrl:eGFP)^la116^* embryos which express GFP in endothelial cells. Injection of 4 ng of morpholino targeted against the exon 2-intron 2 splice junction (nr2f1a^e2i2^ MO) resulted in two obvious vascular phenotypes. Loss of *nr2f1a* showed ISV growth defect and mis-pattern plexus at CVP (caudal vein plexus) (Figure 3 C–H). At 30 hpf, a highly penetrate stalling of ISV growth was observed at the mid-somite at the midpoint of angioblast migration from the DA to the DLAV (Figure 3D, F) with only 30% of complete ISVs (n = 34 embryos) in *nr2f1a^e2i2^* morphants compared to 80% of complete ISVs in uninjected controls (n = 26 embryos). The second phenotype we observed was the disruption of the honeycomb structure in the CVP as compared to uninjected controls at 30 hpf (Figure 3E, F). At 48 hpf, the swallowed plexus even became obvious (Figure 3H). Quantification of loop formation at CVP showed a 4-fold decrease in *nr2f1a* morphants (n = 10 in wt and in nr2f1aMO) at 48 hpf. During zebrafish embryonic angiogenesis, ISV and CVP formation were the two obvious vessel patterns to be observed. ISV angiogenesis is largely controlled by VEGF and Notch signalling. Recent studies showed that endothelial cells are also sprouting from the axial vein ventrally to form the CVP via BMP signalling pathways. Our data indicated that nr2f1a may play a critical role in controlling endothelial tip cell behaviors and in regulating ISV and CVP formation during angiogenesis.

**Figure 4:**
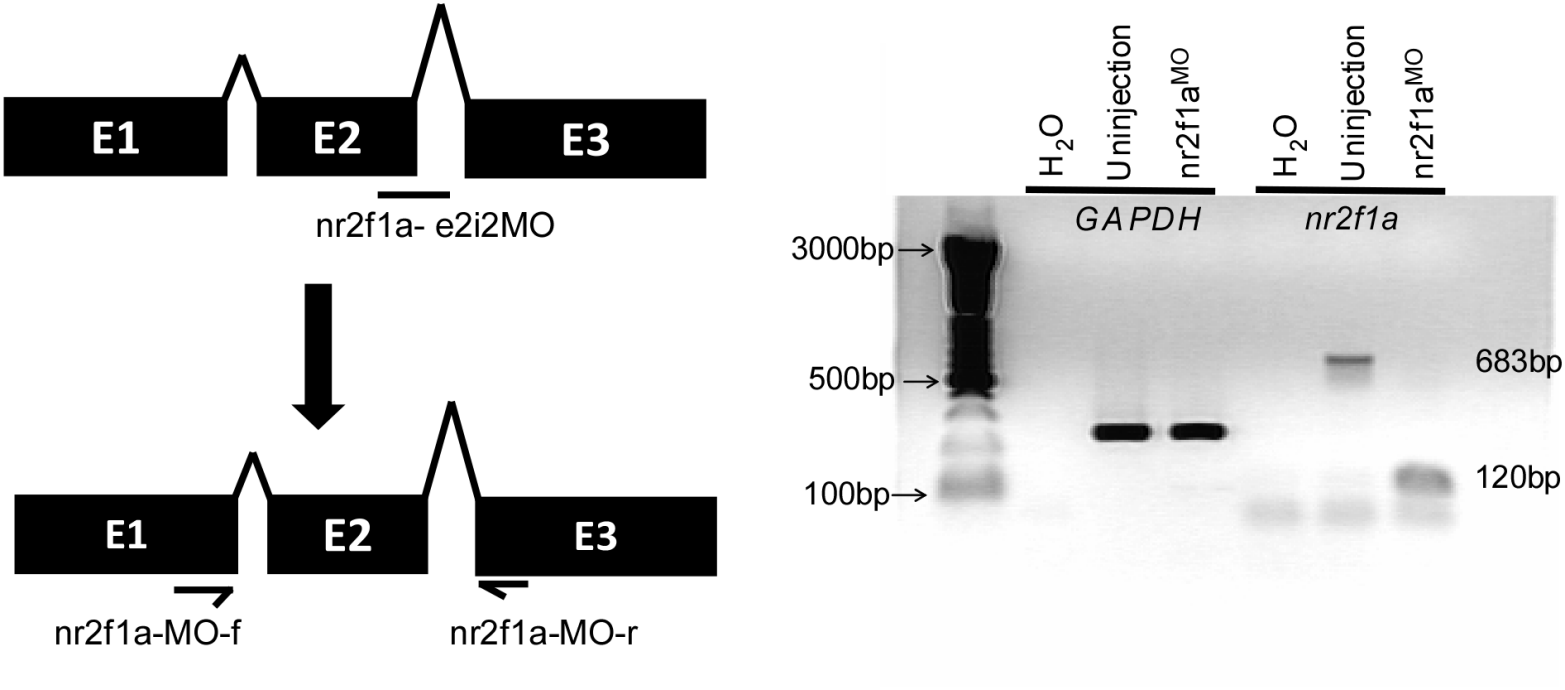
Morpholino-knockdown efficiency of *nr2f1a* in zebrafish embryos. (**A**) Scheme shows nr2f1a morpholino targeting the pre-mRNA structure of nr2f1a and suggested mis-splicing fragment which can be detected by a nr2f1a_MO_f and nr2f1a_MO_r primer set. (**B**) cDNA from uninjected controls or *nr2f1a* morphants (injected with 4 ng morpholino) underwent PCR with primers for the housekeeping control gene (*GAPDH*), or for the *nr2f1a* gene. Morphants injected with nr2f1a^e2i2^, *GAPDH* levels are unchanged while the amount of wild-type *nr2f1a* product (683 bp) got diminished and a new lower molecular weight band appeared (120 bp), representing mis-splicing caused by the morpholino.

We further tested the efficiency of nr2f1a^e2i2^ morpholino knockdown. Injection of 4 ng of nr2f1a^e2i2^ morpholino completely disrupted normal splicing of *nr2f1a* as determined by RT-PCR (Figure 4). To test the specificity of *nr2f1a* morpholino targeting, we analyzed the *nr2f1a* splicing morpholino sequence (nr2f1a^e2i2^ MO) by nucleotide BLAST and showed high specificity (E-value = 10^−5^) compared to a 2^nd^ target (E-value = 2.3). In addition, the sequence comparison to *nr2f1b* and *nr2f2* showed only 36% identity and 44% identity, respectively, suggesting the morpholino targeting to *nr2f1a* is specific (Figure S1A in File S1). Moreover, injection of nr2f1a^e2i2^ morpholino completely disrupted the amount of the *nr2f1a* product, but *nr2f1b* and *nr2f2* levels were unchanged (lane 5–8) compared to uninjected controls as determined by RT-PCR, indicating the specificity of the morpholino knockdown of *nr2f1a* (Figure S1B in File S1).

### Loss of *nr2f1a* results in pericardial edema and defective circulation

**Figure 5:**
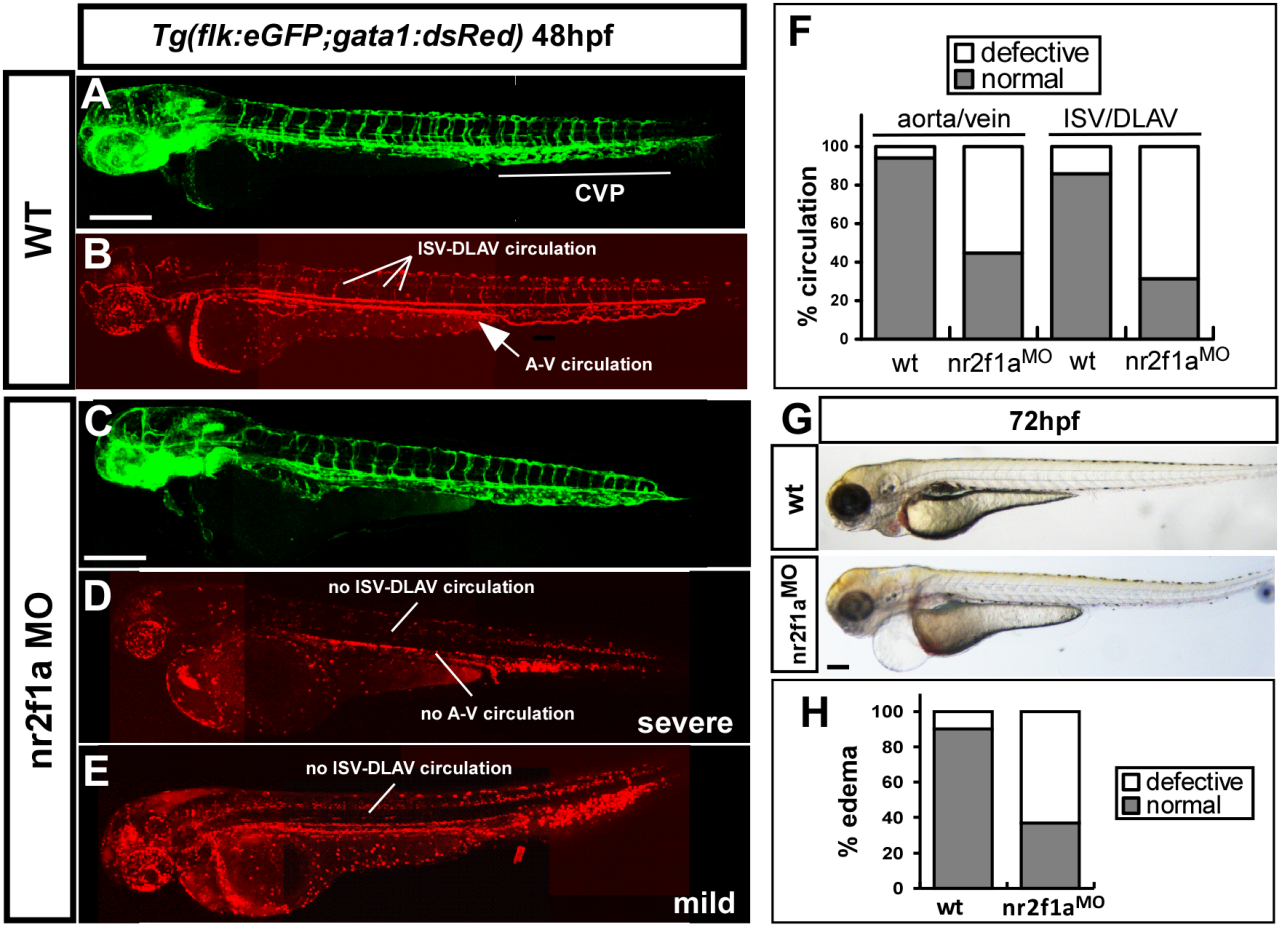
Loss of *nr2f1a* results in circulation defects and pericardial edema. (**A–E**) *nr2f1a* ^e2i2^ MO was injected into transgenic *Tg (fli:eGFP^y1^; gata1:dsRed)* embryos with GFP-labeled endothelial cells (**A, C**) and dsRed-labeled blood cells (**B, D, E**). Loss of *nr2f1a* showed a mis-pattern at ISV and at the caudal vein plexus (CVP) and results in circulation defects in severe (**D**, no circulation and blood stock) or mild (**E**, blood stock at CVP and shorter circulation) at 52 hpf compared to wild-type fish (**B**). (**F**) Circulation defects at the ISV/DLAV (∼68% in morphants) and slow to lose axial circulation of the aorta/vein in the trunk region (∼58% in morphants) are quantitated in wt (n = 37) and nr2f1aMO (n = 42) at 48–52 hpf. (**G, H**) Representative edema fish and quantitative results from three independent experiments showed 65% of *nr2f1a* morphants (n = 69) with mild to severe pericardial edema compared to wt (n = 30). The scale bar in A–E represent 200 µm and in G is 500 µm.

Edema and lack of circulation are common secondary consequences of defective blood vessel formation. We found that a loss of *nr2f1a* causes an increasing pericardial edema. Embryos showed ∼60% mild to severe edema at 72 hpf (n = 30 in wt and n = 69 in *nr2f1a* morphants) (Figure 5G–H). While we examined the circulation in wild-type and morphants, we noted deficient circulation in the trunk of *nr2f1a* morphants at 48 hpf (Figure 5A–F). *nr2f1a* morphants showed less than 50% of embryos with circulation in the axial vessels and less than 40% of the embryos with ISV circulation (n = 37 in wt and n = 42 in *nr2f1a* morphants) (Figure 5F). These data are consistent with the vasculature defects in the *nr2f1a* deficient embryos.

### Phenotypic specificity of the *nr2f1a* morpholino knockdown

We showed knockdown of *nr2f1a* results in vascular defects (Figure 3) and determined the efficiency of the *nr2f1a^e2i2^* morpholino knockdown (Figure 4). To test whether phenotypic specificity of the *nr2f1a* morpholino knockdown, we tested the effects of an additional morpholino targeting the translation initiation site (nr2f1a^ATG^ MO). Our results showed nearly identical vascular phenotypes as *nr2f1a^e2i2^* morphants, including defects in ISV growth (70% decrease), ISV cell number, CVP formation (3-fold decrease), blood circulation and the development of edema occurrence (60% higher) (Figure S2 in File S1), suggesting that the phenotype of *nr2f1a* knockdown is gene-specific.

**Figure 6:**
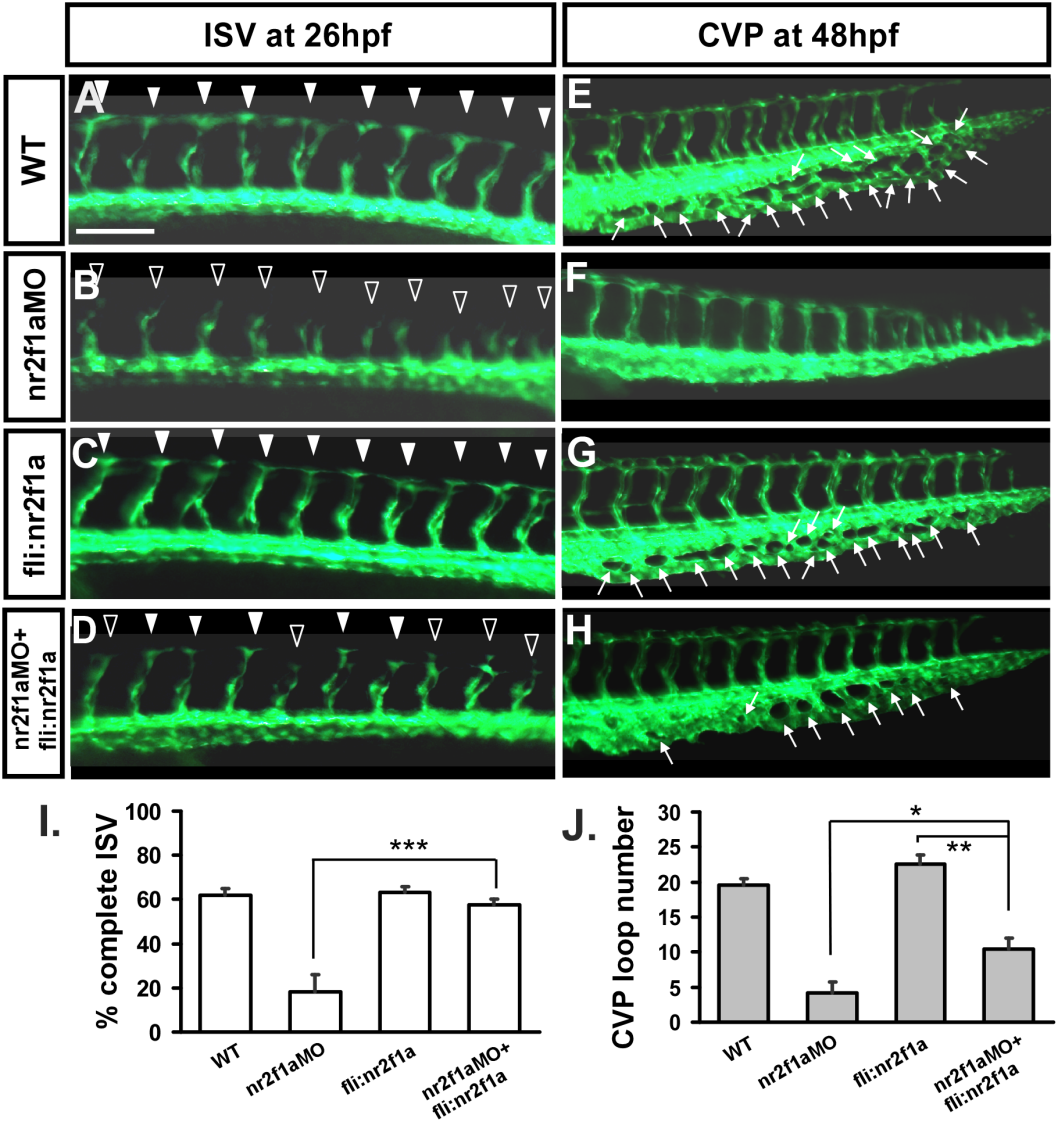
Knockdown of nr2f1a can be rescued by overexpression of *nr2f1a*. In uninjected control embryos, intersegmental vessels (isv) have reached the DLAV at the dorsal region from 24–30 hpf (**A,** *white arrowheads*) and caudal vein plexus (cvp) were formed honeycomb-like structures at the tail around 48 hpf (**E,** *white arrows*). At the same stage, ISVs are stalled at mid-somite (**B,** *hollow arrowheads*) and less/no honeycomb structure is formed at CVP (**F** with *no arrows*) in nr2f1a^e2i2^ morphants. Overexpression of nr2f1a driven by fli promoter had no obvious defect in vasculature (**C, G**), but rescued the defect of ISV stalling (*solid arrowhead*) (**D**) and restored the honeycomb structure at CVP (**H**). (**I**) Quantification of percentage of completed ISV at 26 hpf shows a ∼40% increase in rescued embryos compare to *nr2f1a* morphants. Wild-type embryos had 62±3.1% complete ISV; *nr2f1aMO* had 18±8.0% complete ISV; *fli:nr2f1a* overexpression embryos had 63±2.6% complete ISV and rescued embryos had 57±2.7% complete ISV. (**J**) Quantification of loop formation at CVP showed a 2-fold increased in rescued embryos compared to *nr2f1a* morphants at 48 hpf. Wild-type embryos (19.6±0.9); *nr2f1aMO* (4.2±1.5); *fli:nr2f1a* overexpression embryos (22.6±1.3) and rescued embryos (10.6±1.6). (*** refers to p<0.0001, ** refers to p<0.001 and * refers to p<0.05 by an unpaired student’s t-test. Data are represented as means ± SEM). Scale bars are 100 µm for A–H.

To further confirm the specificity of our morpholino experiments, we performed rescue experiments by overexpression of *nr2f1a* in wild-type and nr2f1a^e2i2^ morphant embryos. Transient transgenic overexpression of *nr2f1a* in endothelial cells using the *fli1* promoter rescues ISV stalling by 40% in *nr2f1a* morphants compared to injection of *nr2f1a* morpholino alone (Figure 6A–D, I), while overexpression of *nr2f1a* in wild-type embryos has no obvious effect on the vascular development (Figure 6C). In addition, overexpression of *nr2f1a* restores the honeycomb-like structure in *nr2f1a* morphants for 50% at CVP (Figure 6H) compared to the *nr2f1a* morphants (Figure 6F). Finally, there was a slightly, but not significant increase in ISV completion and the CVP loop formation when overexpressing *nr2f1a* (Figure 6C, G) suggesting that *nr2f1a* is not sufficient for ISV and CVP growth, at least overexpression driven by *fli* promoter.

### *nr2f1a* is required for the growth of ISV cells

**Figure 7:**
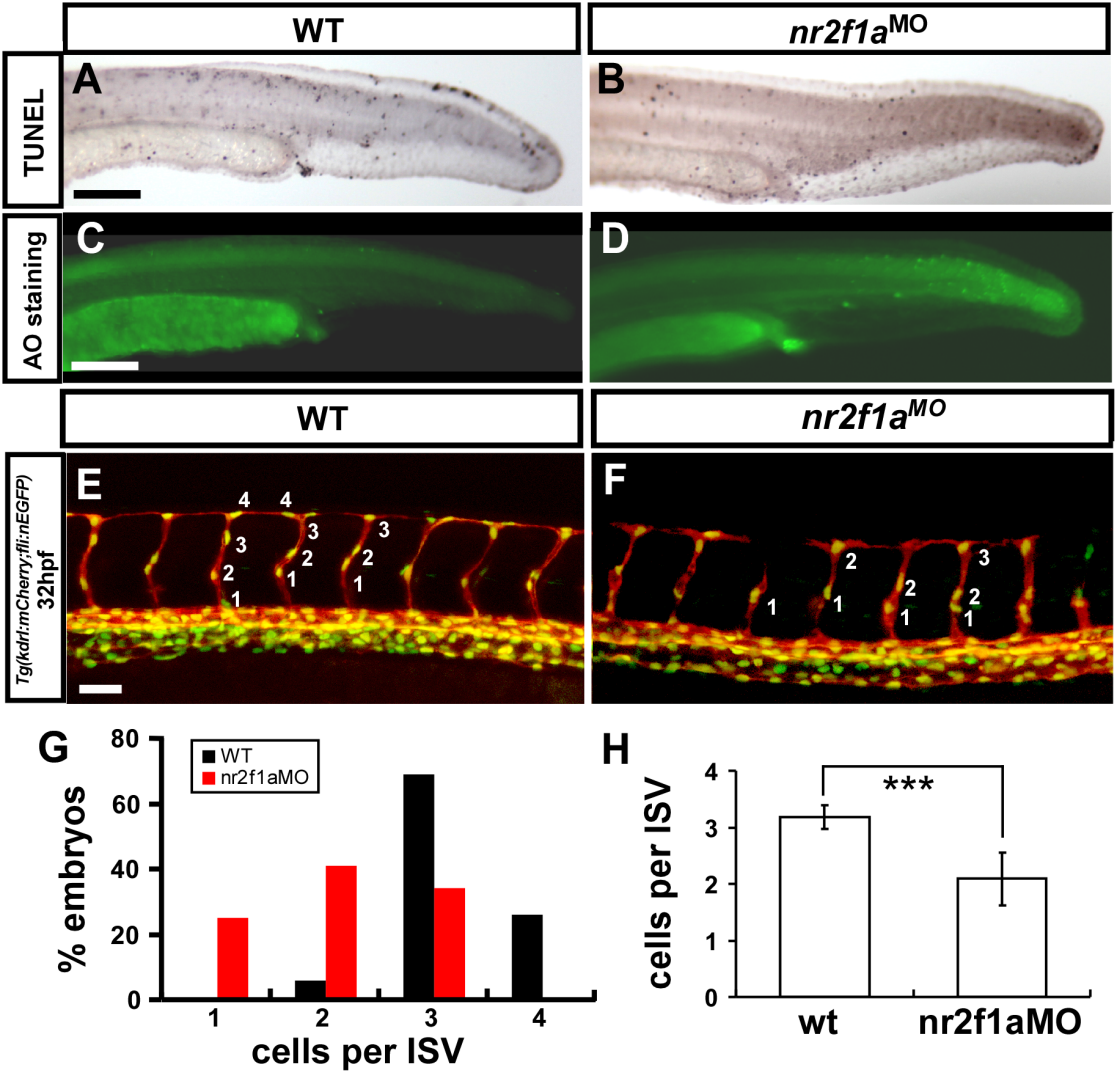
*nr2f1a* is required for the growth of ISV cells. (**A–D**) TUNEL labeling and AO staining was used to detect apoptotic cells in wild type, and nr2f1a^e2i2^ morphants. Although some apoptotic cells were observed on the skin, cell death in vascular regions was not elevated compared to wild type controls. (**E–H**) The number of cells forming each ISV counted in wild type control *Tg(kdrl:mCherry; fli1a:negfp)^y7^* (**E**) and *nr2f1a* morphant embryos (**F**) at 32 hpf. (**G**) Proportional distribution of ISVs containing 1–5 cells and (**H**) average ISV cells per ISV counted in both wild type and *nr2f1a* morphant. (*** refers to p<0.0001 by an unpaired student’s t-test).

Loss of *nr2f1a* results in vascular growth defects suggests the impairment of endothelial cellular migration, proliferation or increase of cell death. To test these hypothesis, we first examine if more cell death in *nr2f1a* morphant? We showed *nr2f1a* morphant phenotypes do not result from morpholino- induced non-specific cell death as there is no significant increase in apoptosis in the trunk of morphants compared to wild type embryos by Acridine Orange staining and TUNEL assay (Figure 7). To test if loss of *nr2f1a* would decrease cell proliferation, we counted the numbers of endothelial cells per ISV in the *Tg (kdrl:mCherry; fli1a:negfp)^y7^* embryos, where GFP was expressed in the nuclear of endothelial cells and the mCherry tag on endothelial ISV cells. Loss of *nr2f1a* showed significantly reduced ISV cells compared to uninjected wt in the control (n = 34 in *nr2f1a* morphants and n = 33 in wt control, *p*<0.0001). Those data suggested that *nr2f1a* was required for ISV cell growth to contribute to the vascular development, likely by regulation of the proliferation or migration of the cells.

### *nr2f1a* modulates vascular identity

**Figure 8:**
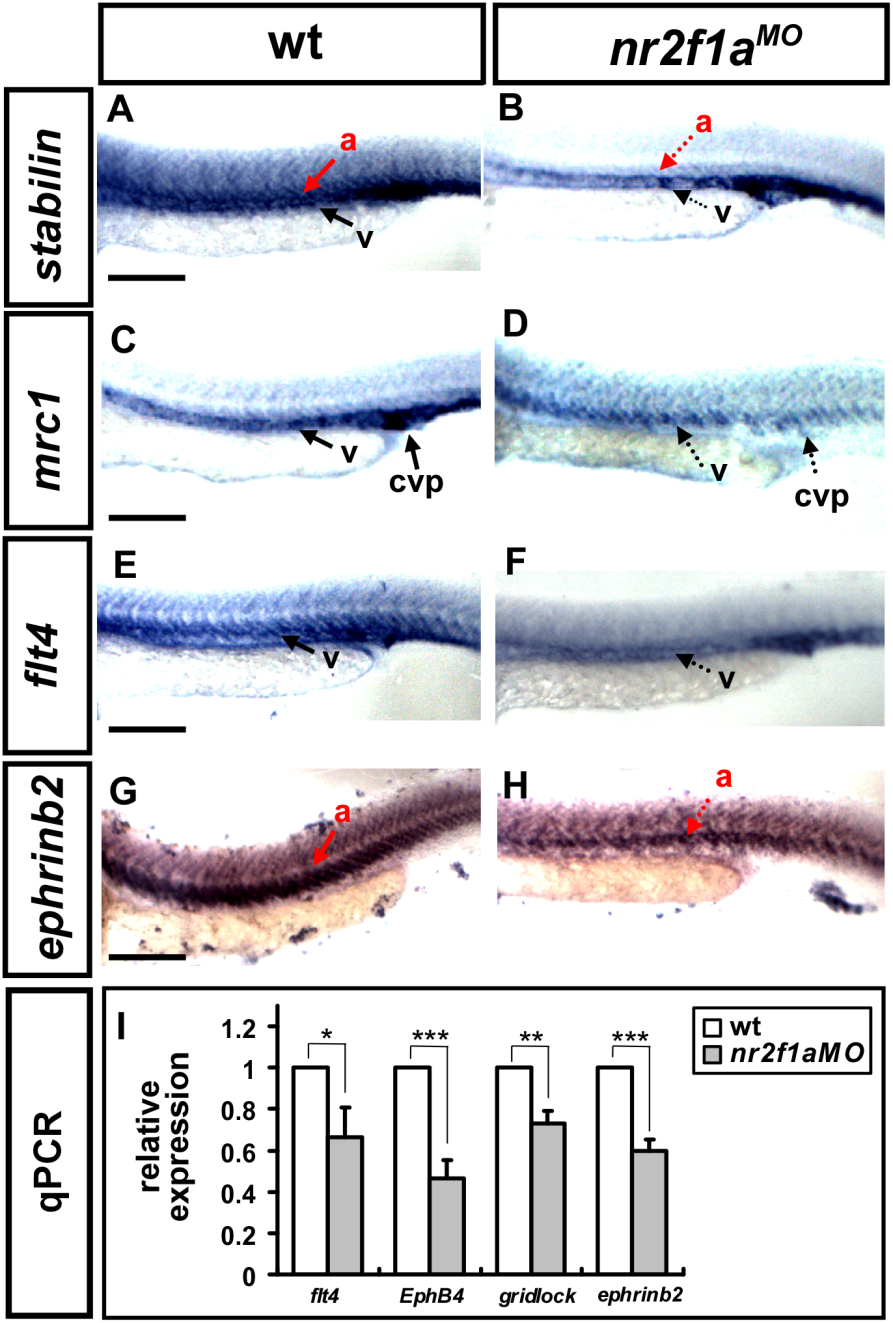
*nr2f1a* modulates the expression of vascular markers. (**A–H**) Compared to wild type controls (A, C, E, G), expression of the venous markers *mrc1* (**D**) and *flt4* (**F**) were reduced in the trunk of *nr2f1a* morphants at 24 hpf. In addition, expression of the arterial marker *ephrinb2* (**H**) and pan-vascular marker *stabilin* decreased (**B**). (**I**) Quantification of the relative expression level by qPCR assay showed a ∼25% to 50% reduced expression in vascular markers *flt4* (0.66±0.14), *EphB4* (0.46±0.08), *gridlock* (0.73±0.06) and *ephrinb2* (0.59±0.05) in *nr2f1a* morphants. *** refers to p<0.0001, ** refers to p<0.001 and * refers to p<0.05 by an unpaired student’s t-test. Data are represented as means ± SEM. Scale bars represent 100 µm in A–H.

ISV growth defect and CVP mis-pattern suggested that *nr2f1a* is important for vascular development and likely modulates vascular identity. To test this hypothesis, we examined the expression of several vascular markers *flt4, mrc1, stabilin,* and *ephrinb2* by in-situ hybridization. We found that the expression of the venous/ISV markers *flt4* and *mrc1* was decreased in *nr2f1a* morphants compared to the wild type controls at 24 hpf (Figure 8). The expression of arterial marker *ephrinb2* and vascular marker *stabilin* are also reduced. To determine the extent of decreased marker expression, we quantified *flt4*, *ephrinb2, ephrinb4 (EphB4)* and *gridlock* transcript levels by qPCR and identified a 20–50% decrease in expression in *nr2f1a* morphants. These results suggest that *nr2f1a* regulates several vascular genes to promote vessel formation.

### Regulatory relationships between *nr2f1a* and *Notch*

**Figure 9:**
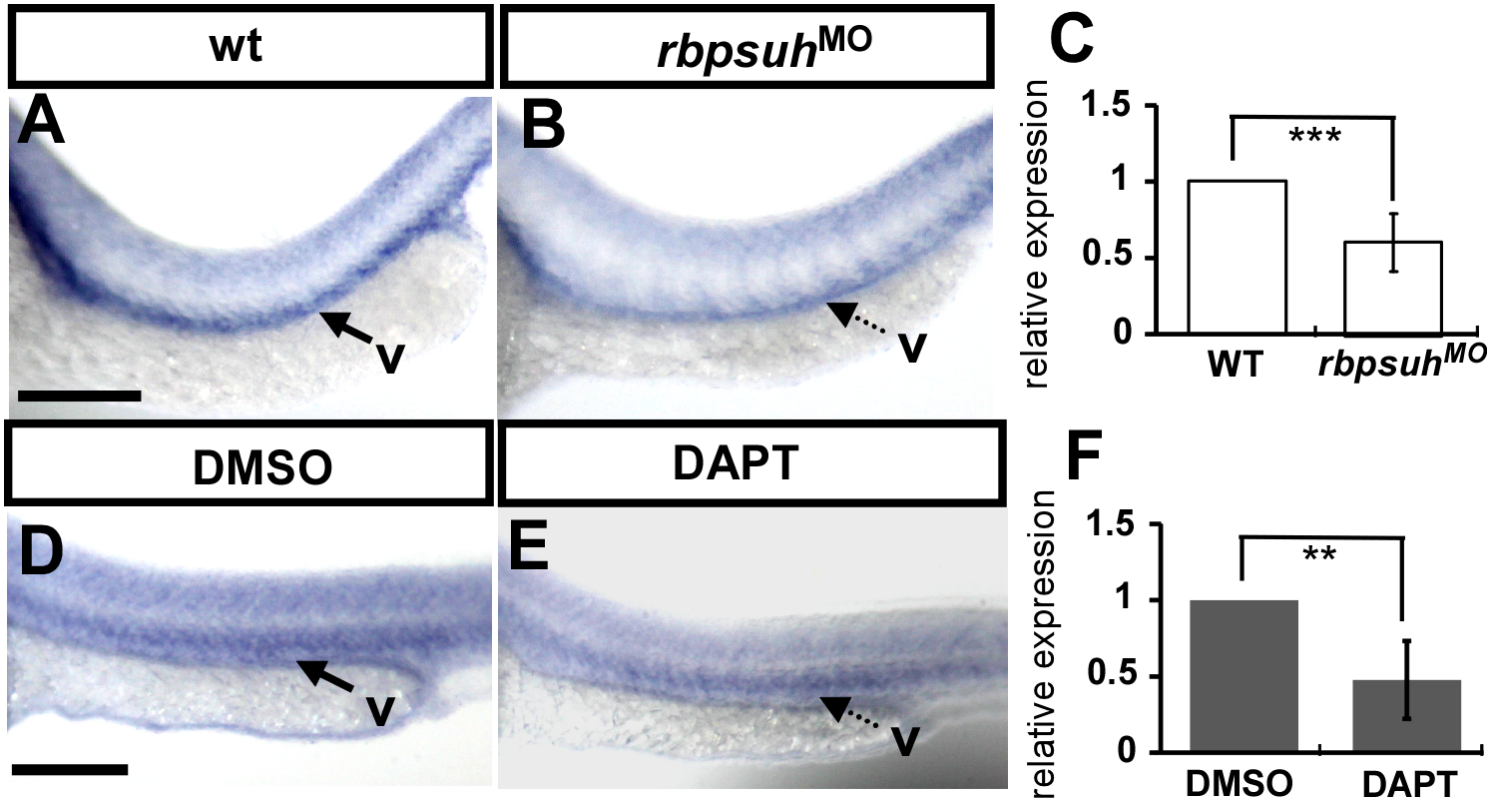
Regulatory relationships between *nr2f1a* and Notch. (**A–B**) *nr2f1a* expression is downregulated in *rbpsuh* morphants (**B**) as compared to untreated control embryos (**A**). (**D–E**) *nr2f1a* expression is downregulated after treatment with DAPT (**E**) as compared to DMSO control embryos (**D**). (**C, F**) Quantification of the relative *nr2f1a* expression level by real-time qPCR assay shows a ∼50% reduced expression in *rbpsuh* morphants (**C**) and DAPT-treated embryos (**F**) compared to uninjected embryos and DMSO-treated controls. Scale bars represent 100 µm in A, B, D and E.

We demonstrated that interruption of *nr2f1a* expression by morpholino injection results in ISV stalling at the midline with a decrease in the number of cells per ISV. In addition, loss of *nr2f1a* causes the deformation of CVP. Notch signalings has been shown to be important for vein identity and the interaction of ISV tip cell with VEGF signaling during angiogenesis. Activation of Notch signaling provides ISV stalling at the midline, while loss of notch signaling leads to an increasing number of cells per ISV [7]. Thus, we hypothesize that inactivation of Notch signalings will increase *nr2f1a* expression. To test if *nr2f1a* interacts with notch signalings, we suppressed Notch signaling by rbpsuhMO injection (Figure 9A–C) or DAPT treatment (Figure 9D–F). Surprisingly, we found *nr2f1a* expression is downregulated when Notch signals were inhibited, with a 50–60% reduction by using in-situ hybridization and qPCR (Figure 9). These data suggest upon the genetic interaction between *nr2f1a* and Notch signals. However, the results contradict our hypothesis that Notch inactivation negatively regulated *nr2f1a* expression. Other regulators that promote angiogenesis, thus, may mask or dominate the function of *nr2f1a* during the Notch inactivation.

## Discussion

In the present study we examined the role of nr2f1a in angiogenesis in vivo during zebrafish development. We first showed nr2f1a is conserved among the vertebrates (Figure 1) and its expression in the developing vessels in zebrafish (Figure 2). We found that knockdown of zebrafish *nr2f1a* by morpholinos led to growth impairment of intersegmental vessels (ISV) and defects in the angiogenic sprouting and formation of CVP (Figure 3), which results in the pericardial edema and circulation defect (Figure 5). TUNEL assay and ISV cells examination suggested vascular defect in *nr2f1a* morphants was associated with impaired proliferation and/or migration of ISV tip cells (Figure 7). Furthermore, genetic evidence suggested that *nr2f1a* interact with Notch signalings (Figure 9). Taken together, these results demonstrate zebrafish nuclear receptor nr2f1a ortholog to mammalian coupTFI/II is essential for vascular patterning *in* *vivo*. The cooperative function and combinatory effects between *nr2f1a* and its paralog *nr2f1b* and *nr2f2* in zebrafish will be tested in the future.

The angiogenic sprouting of zebrafish ISVs and CVPs is regulated by different signaling mechanisms and endothelial cells respond to angiogenic cues is critical to coordinate distinct cellular behaviors to form an organized vessel network. Molecular mechanisms that transcription factors promote angiogenesis of ISV and CVP are still not well-understood. To reveal the molecular mechanisms that control vascular patterning, we first focused on the roles transcription factors play in vascular patterning. We previously identified LIM-transcription factor islet2 functions in the specification of venous and tip cells in zebrafish (unpublished results). Other research groups and we also found that in zebrafish *nr2f2* plays only a minor role in early vascular development, but have a significant function in venous angiogenesis and lymphogenesis [12], [28], [29] and our unpublished results. In this study, we emphasize the role *nr2f1a* has in ISVs growth and CVP sprouting and extension. It is likely involved in cell proliferation and migration during the angiogenic process. Meanwhile, our data suggested that *nr2f1a* likely affects ISV and CVP angiogenesis directly because we found *nr2f1a* mainly expressed in vascular derivatives and no mural cells are present at the early stages of development.

ISVs cells are derived from angioblasts that are sprouting from the dorsal aorta and vein. Stalling of intersegmental vessel growth at the mid somite might, therefore, either occur through defective proliferation or defective migration of cells. We here demonstrated that a knockdown of *nr2f1a* in zebrafish leads to ISV growth stalling and a decreased number of ISV cells. TUNEL analysis suggests that cell death is not increased in the trunk region, suggesting that *nr2f1a* is necessary to promote a proliferation of ISV cells. However, the ultimate fate of these cells as tip or stalk cells remains unknown.

The process of ISV formation is largely controlled by VEGF and Notch signaling [4]. In this study we test the interaction between *nr2f1a* and Notch signaling. Surprisingly, we observed positively regulation between Notch signaling and *nr2f1a* expression. These data suggest on a genetic interaction between *nr2f1a* and Notch signals. However, these results contradict to our hypothesis that Notch inactivation negatively regulated *nr2f1a* expression. Thus, other regulators that promote angiogenesis mask or take over the function of *nr2f1a* during the Notch inactivation. On the other hand, the requirement of *nr2f1a* for vascular integrity maybe mediated by other signals. It would be interesting if other signaling molecules, such as Wnt/β-catenin or BMP/smad interact with *nr2f1a*. We will address this question in the future.

As for our previous findings and our current study, we would like to ask whether zebrafish *nr2f1a* plays a conserved role in vasculature similar to *Nr2f2* in mice? Our previous study suggested that *nr2f2* plays a minor role in vein and tip cell differentiation but acts as a major determinant of lymphogenesis in zebrafish (unpublished results). In this study, we showed that zebrafish *nr2f1a* is the ortholog to mouse Nr2f2 and plays a role in ISV and CVP angiogenesis, which is consistent with the partial function of Nr2f2 in mice [11]. Those data indicate the conserved vascular function of the NR2F family among vertebrates. Our phylogenetic analysis of NR2F members among vertebrates suggests that zebrafish nr2f1a and nr2f2 are closer to mammalian nr2f2. On the other hand, zebrafish nr2f1b is much different from the zebrafish paralogs nr2f2/nr2f1a but evolutionary conserved to its ortholog mammalian Nr2f1. The function of mouse Nr2f1 has been identified and the transcriptional targets of Nr2f1 are identified in inner ear tissue of mice [31]. Their gene ontology categories are cell adhesion, cartilage development, cell cycle regulation, myeloid cell differentiation and lipid/steroid metabolism. Thus, there is so far no evidence showing a mammalian Nr2f1 function in vasculature. Since mice Nr2f1 is a critical regulator for neural development and controls cell differentiation in the inner ear, it would be interesting to test whether zebrafish nr2f1a has other functions that might be similar to mammalian Nr2f1. The same holds for zebrafish nr2f1b which might have functions similar to mammalian Nr2f1.

In conclusion, we demonstrate a new transcription factor nr2f1a participate in ISV growth, and reveal a novel role of nr2f1a in vascular biology. Finally, to understand the molecular mechanisms that *nr2f1a* control the pattering of ISV and CVP, transcriptome analysis of *nr2f1a* downstream targets could be very interesting and will be pursued in the near future. The potential downstream targets of *nr2f1a* will be interesting to characterize and may be served potential targets to suppress angiogenesis, offering important therapeutic implications, such as cancer therapy.

## Supporting Information

File S1 **Combined Supporting Information file.**
(DOC)
