# Supplementary material for: Nuclear Receptor Subfamily 2 Group F Member 1a (nr2f1a) Is Required for Vascular Development in Zebrafish
Source: PLoS One. 2014 Aug 26;9(8):e105939. doi: 10.1371/journal.pone.0105939 (PMC4144922; doi:10.1371/journal.pone.0105939)
Supplement: File S1 — Combined Supporting Information file. (DOC) [file pone.0105939.s001.doc]

**Table S1: Primer sequences for qPCR experiments**

| **qPCR primers** | **Sequence** |
| --- | --- |
| EF1α_2f | 5’-TCAACGCTCAGGTCATCATC-3’ |
| EF1α_2r | 5’-GATGTGAGCAGTGTGGCAATC-3’ |
| flt4_f1 | 5’-ACTCGGGTTATTACCGCTGCTTCT-3’ |
| flt4_r1 | 5’-TGGATGCTCTGGGTCTCGAACAAA-3’ |
| gridlock_f2 | 5’-CCCAAGTCATGGCCAGAAAG-3’ |
| gridlock _r2 | 5’-GACGACGCAACTCTGATAAGC-3’ |
| EphB4_f | 5’-GGTGTCCGACTTTGGTCTGT-3’ |
| EphB4_qr | 5’-CCAGCGGATGGGAATTTTAC-3’ |
| ephrinb2a_f | 5’-GGTTCAATGGAAGGAGTGGA-3’ |
| ephrinb2a_qr | 5’-AGAGGCGTGTCTGCTTTTGT-3’ |
| nr2f1a_f | 5’-TTTTCGCTGGACCAGATGAG-3’ |
| nr2f1a_qr | 5’-AGTTGGCATGAGACGGGAAG-3’ |


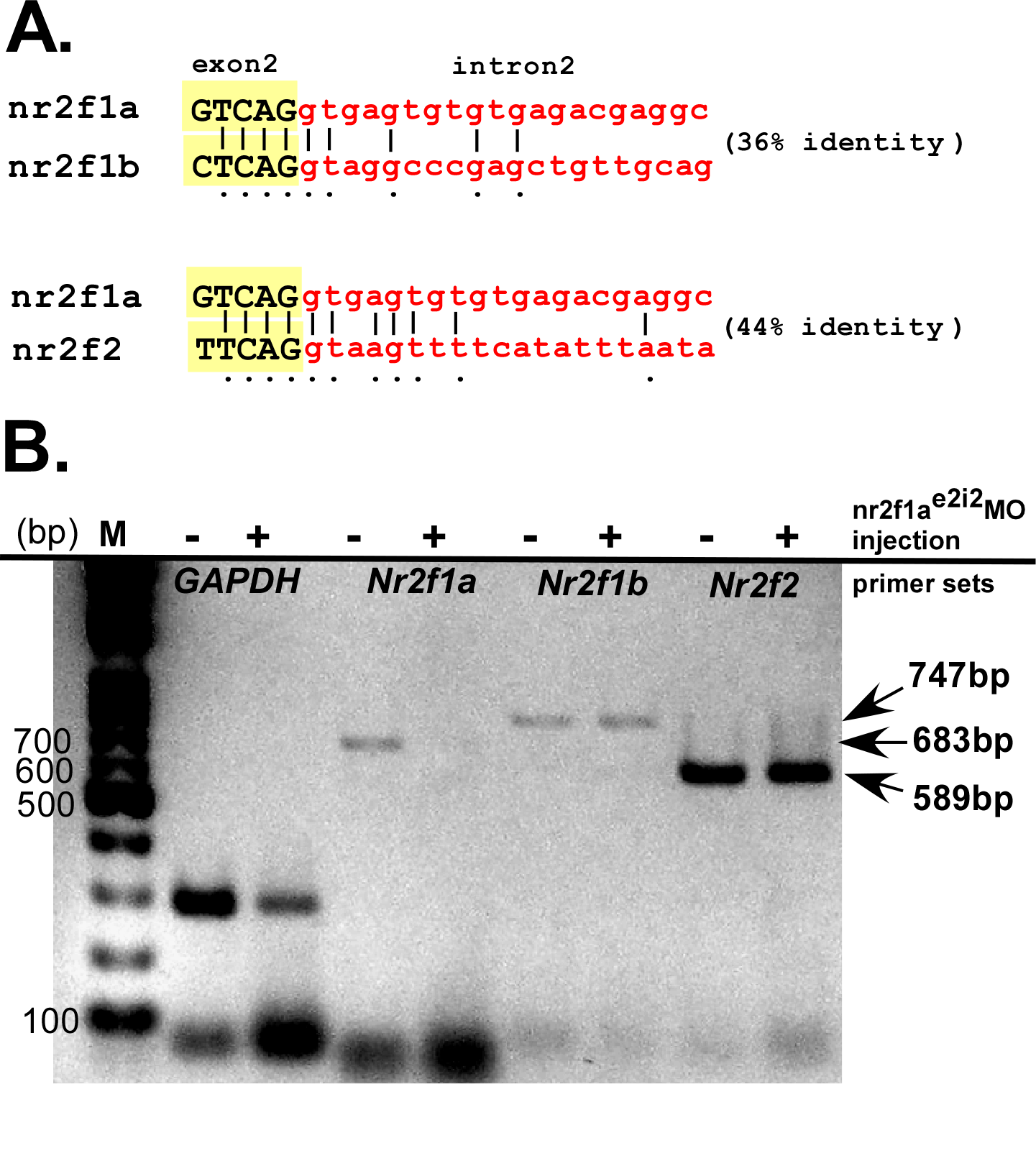


**1 2 3 4 5 6 7 8**

**Figure S1. Specificity of nr2f1a morpholino targeting.**

(A) Nr2f1a splicing morpholino sequence (nr2f1a^e2i2MO^) design was targeted against the exon2-intron2 splice junction. The sequence comparison to nr2f1b and nr2f2 are only 36% identity and 44% identity, respectively, suggesting the morpholino targeting to nr2f1a is specific. (B) cDNA from uninjected controls (-) or nr2f1a^e2i2MO^ injected embryos (+) underwent PCR with primer sets of the control gene *GAPDH*, *nr2f1a*, *nr2f1b* and *nr2f2.* In morphants injected with nr2f1a^e2i2^ morpholino, *nr2f1b* (747bp) and *nr2f2* (589bp) levels are unchanged (lane 5-8) while the amount of *nr2f1a* product (683bp) is diminished (lane 3-4) compared to uninjected controls, indicating the morpholino knockdown of *nr2f1a* is specific.


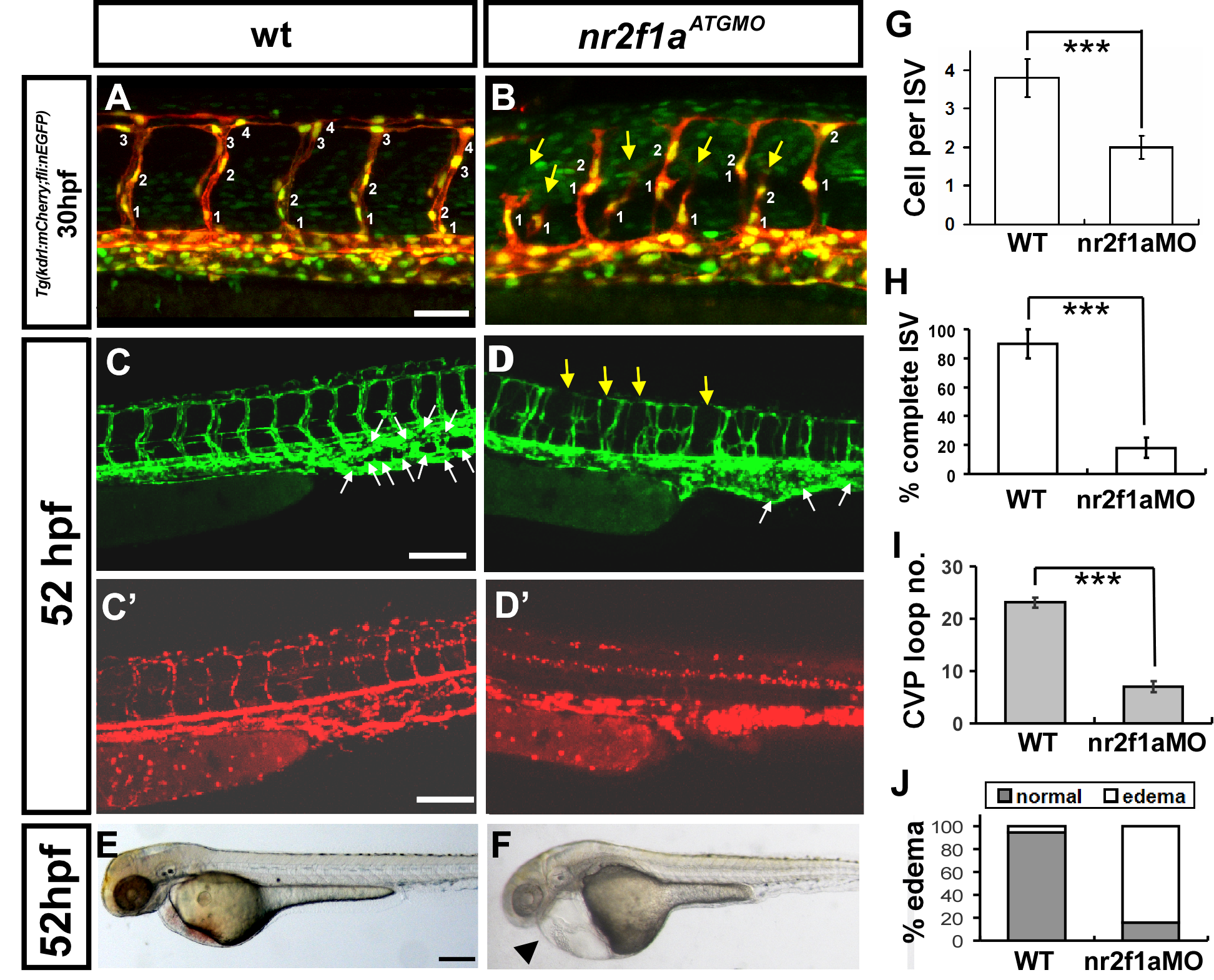


**Figure S2. Knockdown of nr2f1a causes defects in vascular development in zebrafish by using translational block ATG morpholino**

(A-D) Loss of *nr2f1a* showed ISV growth defect (yellow arrows in B and D) and mis-pattern plexus at CVP (caudal vein plexus) (white arrows in D) at 30hpf and 52hpf. At 30 hpf, in uninjected control embryos, intersegmental vessels (ISV) have reached the DLAV of the embryo (A) and caudal vein plexus were formed honeycomb-like structures at the tail (white arrows in C). At the same stage ISVs are stalled mid-somite in nr2f1a^ATG^ morphants (B, D). (G) The number of cells forming each ISV were counted from wild type control *Tg(kdrl:mCherry; fli1a:negfp)^y7^* and *nr2f1a* morphant embryos at 30 hpf, showing a significant decrease in *nr2f1a* morphant. (H) Quantification of percentage of completed ISV shows a ~70% increase compare to *nr2f1a* ATG morphants (n=24 in wt and n=20 in nr2f1aMO) at 30 hpf. (I) Quantification of loop formation at CVP at 48-52hpf shows a ~4-fold decreased in *nr2f1a* morphants (n=8 in wt and n=12 in nr2f1aMO). In addition, loss of *nr2f1a* results in circulation defects (blood stock at vessel/CVP or no circulation at ISV/DLAV region) (D’) and pericardial edema (arrowhead in F) shown at 52hpf. (J) Quantitative results showed ~80% of *nr2f1a* morphants (n=18) with pericardial edema compared to wt (n=20). (*** refers to p< 0.0001 by an unpaired student's t-test. Scale bars are 50 μm for A-B, 100 μm for C-D, C’-D’, and 200 μm for E-F.
